# Nocturnal systolic blood pressure pattern of type 2 diabetic hypertensive men with erectile dysfunction: a cross sectional study from Northern Sri Lanka

**DOI:** 10.1186/s13104-019-4740-z

**Published:** 2019-10-25

**Authors:** Thirunavukarasu Kumanan, Vathulan Sujanitha, Nadarajah Rajeshkannan, Balasingam Nisahan

**Affiliations:** 1 Department of Medicine, Faculty of Medicine, University of Jaffna, Jaffna, Sri Lanka; 2 Civic Park Medical Centre, Sydney, NSW 2145 Australia; 3 Teaching Hospital, Jaffna, Sri Lanka

**Keywords:** Nocturnal blood pressure pattern, Erectile dysfunction, Diabetes, Northern Sri Lanka

## Abstract

**Objective:**

This small scale cross-sectional study was done to identify the common systolic blood pressure pattern (dipping or non-dipping) among type 2 diabetic men with coexisting hypertension and erectile dysfunction(ED). A recent study in the same setting showed that prevalence of ED was high among diabetic men and co-existing hypertension was identified as an independent risk factor. There was a postulation about an association between ED and non-dipping nocturnal blood pressure pattern. So ambulatory blood pressure measurements (ABPM) was obtained for participants to test this prediction. Data was analysed using SPSS 25 Version.

**Results:**

Among 29 participants who underwent ABPM, 21 showed non-dipping pattern of nocturnal systolic blood pressure (72.4%; CI 54.3–86.3). Mean SBP of participants was 125.55 +_14.1 and Mean DBP was 81.5 + _12.82. There was no statistical difference observed in mean SBP and DBP between patients with dipping nocturnal SBP and non-dipping pattern (P > 0.05). Variability of SBP was high among the participants (Mean SD-11.96 +/_2.74) and DBP also showed relatively high variability (SD-9.28 +/_2.9). Mean dipping percentage of the SBP during sleep was 5.54 +/_6.66. A significant difference in heart rate (HR) between patients with non-dipping and dipping pattern was noted (P-0.034).

## Introduction

Hypertension is a well-recognized risk factor for development of Erectile Dysfunction (ED). A recent study from the diabetic centre of Northern Sri Lanka from which this study population was selected, revealed that the prevalence of ED among type 2 diabetic men was very high and co-existing hypertension was identified as an independent risk factor for ED [1]. Studies also found that hypertension in a diabetic significantly increases the incidence of atherosclerosis which might affect arteries including penile arteries [2, 3]. Masked hypertension/nocturnal hypertension should always be born in mind among diabetics and patients with chronic kidney disease (CKD). These common unrecognized phenomena have great implications on development of end organ damage and might help in scheduling the antihypertensive medication regimen accordingly. ABPM is nevertheless an invaluable investigation in this regard [4]. And it is a well-known fact that non-dipper status or reverse dipping is common among patients with Parkinson’s disease where autonomic dysfunction is a common association. They typically has either reversal of circadian rhythm, postprandial hypotension or nocturnal hypertension [5–7]. A study in Turkey suggested an association between ED and non-dipping status of hypertension and a common pathway of autonomic dysfunction (sympathetic overactivity) for ED and non- dippers postulated as a pathophysiological explanation [8]. But nocturnal blood pressure pattern (non-dipping status) has not been studied extensively both internationally as well as in this part of the world. This small scale study intended to identify common nocturnal systolic blood pressure pattern among the diabetic men with ED and co-existing hypertension.

## Main text

### Patients and methods

#### Study design participants

This is a cross sectional study among type 2 diabetic men with ED and co-existing hypertension to find out the common nocturnal systolic blood pressure pattern.

Sampling: 30 patients were selected randomly(simple random sampling) from the 113 diabetic men with ED and co existing HT from a previous study [1] which was recently done in the same setting(Diabetic centre of Teaching Hospital Jaffna, Sri Lanka). In the previous study an International Index of Erectile function (IIEF-5) questionnaire was used to evaluate ED [1]. All 30 patients were agreed to undergo (ambulatory blood pressure monitoring) ABPM and 29 of them completed the test during study period.

Study Period: August 2018–September 2018. Extension of the ethical clearance was obtained from ethical review committee, Faculty of Medicine, University of Jaffna (J/ERC/17/88/DR/0054) in July 2018 as part of the recent prevalence study conducted in the same setting.

Operationalization: ambulatory blood pressure monitoring (ABPM) was done by using machine Scanlight III Vers B. ABPM device was programmed to perform the measurement per 30 min in daytime (06:00–22:00 h) and per 60 min in Night time (22:00–06:00 h).

Dipping of SBP pattern was calculated as follows: (1 − (Mean systolic BP in night time/Mean SBP in day time)) × 100. After that participants were classified into dippers if night time SBP dropped more than 10% [4, 9] and if not classified as non-dipping SBP (non-dippers).

Data were analysed by using SPSS version 25.0. Continuous variables are presented as mean ± standard deviation (SD) and for categorical variables percentage calculated with 95% CI. Comparison of means between non-dipping group and dipping group was made using t test and P value of < 0.05 was considered as statistically significant.

### Results

Among 30 type 2 diabetic men with ED and co-existing hypertension, 29 completed ABPM. Mean age of study participants was 51.24 ± 6.04 with minimum age 38 and maximum age 59. Among 29 participants 21 of the diabetic men with ED and coexisting hypertension showed non-dipping pattern of systolic blood pressure(SBP) (72.4%;CI 54.3–86.3%) (Table 1). There was no significant differences observed for mean age between groups showed non-dipping nocturnal SBP pattern and non-dippers (P−0.950). Mean SBP and DBP from ABPM readings of participants were 125.55 and 81.59 respectively. There were no statistical difference observed in mean SBP and DBP between the group showed dipping night time SBP pattern and non-dippers(P > 0.05). Among the 29 participant 4 were (13.8% CI 4.5%30.0%) found to have average SBP above 140 mmHg and 7 participants (24.1% CI 11.2–42.0%) found to have average DBP above 90 mmHg. Mean MAP of the population was 101.66 +/_13.07. Variability of SBP was high among the participants (Mean SD-11.96 +/_2.74) and DBP also showed relatively high variability (SD-9.28 +/_2.9). The variability of both SBP and DBP not showed significant differences between the group showed non-dipping nocturnal SBP pattern and dippers(P value was 0.660, 0.076 respectively). Mean dipping percentage of the SBP during sleep was 5.54 +/_6.66. The group showed dipping night time SBP pattern (dippers)and non-dippers showed significant differences in dipping percentage in SBP while awake and while sleep(P-0.002 and < 0.001 respectively)(Table 2). Study also revealed significant differences in HR between non-dippers and dippers(P− 0.034).

**Table 1:** Basic statistics of participants

Basic statistics	Mean +/_SD or number(percentage with 95% CI)
Mean age	51.24 +/_6.04
Mean SBP (24 h) (mmHg)	125.55 +/_14.10
Mean DBP (24 h) (mmHg)	81.59 +/_12.82
Mean HR	73.66 +/_10.17
Mean MAP	101.66 +/_13.07
Mean SD of SBP	11.96 +/_2.74
Mean SD of DBP	9.28 +/_2.90
Mean dipping percentage while awake	3.14 +/_6.36
Mean dipping percentage while sleep	5.54 +/_6.66
Non-dipper	21 (72.4%; CI 54.3%–86.3%)
SBP (24 h) (mmHg) > 140	4 (13.8%; CI 4.5%–30.0%)
Average DBP (24 h) (mmHg) > 90	7 (24.1%; CI 11.2%–42.0%)

**Table 2:** The statistical comparisons of ambulatory blood pressure monitoring (ABPM) measurements, heart rate variability in patients with Dipper and Non dipper Hypertension

Statistics	Patients with dipping pattern of SBP during sleep N:8	Patients with non-dipping pattern of SBP during sleep N:21	*P* value
Mean age	51.13 +/_5.82	51.29 +/_6.26	0.950
Mean systolic blood pressure (24 h) (mmHg)	123.38 +/_15.66	126.38 +/_13.78	0.617
Mean diastolic blood pressure (24 h) (mmHg)	80.38 +/_13.47	82.05 +/_12.87	0.760
Average HR	67.25 +/_9.85	76.16 +/_9.39	0.034
Average MAP	100.13 +/_14.17	102.24 +/_12.94	0.705
Mean SD of SBP	12.34 +/_4.21	11.82 +/_2.05	0.660
Mean SD of DBP	10.82 +/_3.12	8.69 +/_2.65	0.076
Mean dipping %of SBP during awake	8.62 +/_4.76	1.06 +/_5.66	0.002
Mean dipping %of SBP during sleep	12.76 +/_3.01	2.80 +/_5.50	< 0.001

### Discussion

Although nocturnal hypertension recently gained recognition in the hypertension arena, it is often overlooked during the day to day clinical practice. But it is a well-known factor that non-dipping blood pressure pattern have more hypertension-induced organ damage [10] which make us to postulate patients with erectile dysfunction, type 2 diabetes and coexisting hypertension probably will show a non-dipping nocturnal blood pressure pattern. A previous study done in the same settings has found that ED was very high among diabetic men (62.9%) and particular study also showed that co-existing hypertension is an independent risk factor (AOR: 1.9; 95% CI 1.1–3.19) [1]. A recent study from Turkey concluded that patients who showed “non-dipping” status could share a common autonomic pathway in patients with ED [8]. This again proves that this postulated theory could play a key role in the abnormal blood pressure patterns among ED. But no study to date was done to investigate this hypothesis in a South Asian population. This study was carried out in a resource poor setting and attempted to identify the common pattern of nocturnal systolic blood pressure pattern among diabetic (type 2) men with ED and co-existing hypertension by investigating with a standard ABPM. ABPM is a useful investigation to identify white coat hypertension, masked hypertension, nocturnal hypertension, drug related hypertension, and labile hypertension. However it is still under-utilized in Sri Lanka due to the lack of awareness and unavailability. When considering the blood pressure pattern usual drop in nocturnal blood pressure is normal and which was defined as drop of more than 10% of the diurnal BP values [11] if not BP pattern known as “non-dippers”.

As we expected 21 participants showed “Non- dipping” SBP pattern during sleep (72.4%; CI 54.3%–86.3%). Even though exact mechanism of the nocturnal hypertension is not fully understood studies suggested that increased sympathetic activity and decrease parasympathetic activity throughout the night is a possible explanation [11, 12]. A study done with the objective of testing hypothesis non-dipping nocturnal hypertension more prone to develop erectile dysfunction, proved that non-dipping blood pressure pattern statistically significantly associated with more erectile dysfunction(P = 0.004) and authors concluded sympathetic over activity is possible explanation and both ED and non-dipping blood pressure status sharing common pathological pathway [13]. This findings supports our study findings and also our study showed average HR among participants showed dipping nocturnal SBP pattern and non-dipping SBP pattern that were 67.25 +/_9.8 and 76.16 +/_9.39 respectively. This difference is statistically significant (P− 0.034). This finding further emphasizes an autonomic dysfunction pathological path way (sympathetic over activity).

Non-dippers with essential hypertension is associated with “more advanced left ventricular hypertrophy, left ventricular mass and left ventricular mass index, carotid artery wall thickness, carotid artery atherosclerotic plaques, silent cerebral infarct, stroke, cognitive impairment and micro-albuminuria” [8]. During sexual arousal physiological activation of parasympathetic nerves to penis release acetyl choline which induce the endothelial cells of trabecular sinusoids to secrete nitroxide which activate cGMP(cyclic Guanosine Mono Phosphate) pathway and results in relaxing of smooth muscle and increase the blood supply of penile arteries [14–16]. Mechanism of non-dippers also related to autonomic dysfunction resulting in fall of sympatho-vagal nervous activity [17, 18]. So it is reasonable to assume that the majority of ED with hypertension could be having non-dipping SBP pattern and this small scale cross sectional study has provided some evidence for this. Mean dipping percentage of SBP during awake (P-0.002) and Mean dipping percentage of SBP during sleep (P < 0.001) significantly differed among dippers and non-dippers.

In addition we also noted variability of SBP and DBP was high among the participants as well (Mean SD of SBP-11.96 +/_2.74 and Mean SD of DBP-9.28 +/_2.90 respectively). Other risk factors for ED such as dyslipidemia, BMI, medication adherence, obstructive sleep apnoea, alcohol intake, and smoking are associated with ED [19] and might be having some role in the nocturnal hypertension but it was not explored in our study.

### Conclusion

As we expected diabetic men with ED and co existing blood pressure showed more non-dipping pattern of systolic blood pressure during sleep. Sympathetic over activity could be possible explanation for this findings and the non-dipper status has no correlation with blood pressure variability and the level of blood pressure. Further methodologically well-defined comparative study is recommended to test the hypothesis of non-dipping pattern is more associated with ED.

## Limitations

Above findings need to be interpreted cautiously as we have not done a comparative study instead we just observed among a selected type 2 diabetic men with ED and coexisting hypertension and it showed non-dipping pattern of nocturnal SBP. Due to constrains(only two ABPM machine available for entire hospital and patients were not allowed to go home after fitting BP monitor, as such patients were stayed at hospital for 1 day) of resources this preliminary study is limited to 30 in number. Actual estimation of sample size with a comparison group could improve the validity of the study. Also collection of other covariates and multivariate analysis could have been done to improve the validity of the study. Also worth note that diabetic patients are not routinely screened for ED until our first prevalence study (Reference 1) as such we selected sample from the patients who were identified having ED in previous study.

## Data Availability

Data can be provided on request from corresponding author (VS) or NR.

## Abbreviations

ABPM: ambulatory blood pressure measurements
ED: erectile dysfunction
SBP: systolic blood pressure
DBP: diastolic blood pressure
HR: heart rate
MAP: mean arterial pressure
